# Data hosting infrastructure for primary biodiversity data

**DOI:** 10.1186/1471-2105-12-S15-S5

**Published:** 2011-12-15

**Authors:** Anthony Goddard, Nathan Wilson, Phil Cryer, Grant Yamashita

**Affiliations:** 1 Center for Library and Informatics, Woods Hole Marine Biological Laboratory, Woods Hole, MA 02543, USA; 2 Encyclopedia of Life, Center for Library and Informatics, Woods Hole Marine Biological Laboratory, Woods Hole, MA 02543, USA; 3 Center for Biodiversity Informatics (CBI), Missouri Botanical Garden, St Louis, MO 63119, USA; 4 Center for Biology and Society, Arizona State University, Tempe, AZ 85287, USA

## Abstract

**Background:**

Today, an unprecedented volume of primary biodiversity data are being generated worldwide, yet significant amounts of these data have been and will continue to be lost after the conclusion of the projects tasked with collecting them. To get the most value out of these data it is imperative to seek a solution whereby these data are rescued, archived and made available to the biodiversity community. To this end, the biodiversity informatics community requires investment in processes and infrastructure to mitigate data loss and provide solutions for long-term hosting and sharing of biodiversity data.

**Discussion:**

We review the current state of biodiversity data hosting and investigate the technological and sociological barriers to proper data management. We further explore the rescuing and re-hosting of legacy data, the state of existing toolsets and propose a future direction for the development of new discovery tools. We also explore the role of data standards and licensing in the context of data hosting and preservation. We provide five recommendations for the biodiversity community that will foster better data preservation and access: (1) encourage the community's use of data standards, (2) promote the public domain licensing of data, (3) establish a community of those involved in data hosting and archival, (4) establish hosting centers for biodiversity data, and (5) develop tools for data discovery.

**Conclusion:**

The community's adoption of standards and development of tools to enable data discovery is essential to sustainable data preservation. Furthermore, the increased adoption of open content licensing, the establishment of data hosting infrastructure and the creation of a data hosting and archiving community are all necessary steps towards the community ensuring that data archival policies become standardized.

## Introduction

Today, an unprecedented volume of primary biodiversity data are being generated worldwide [1], yet significant amounts of this data have been and will continue to be lost after the conclusion of the projects tasked with collecting them [2]. Gray *et al*. [3] make a distinction between ephemeral data, which, once collected, can never be collected again, and stable data, which can be recollected. The extinction of species, habitat destruction and related loss of rich sources of biodiversity make ephemeral a significant amount of data that have historically been assumed to be stable. Whether the data are stable or ephemeral, however, poor record keeping and data management practices nevertheless lead to loss of data [4]. As a result, biodiversity data collected today are as endangered as the species they represent. There are also important questions of access to and interpretation of the data. Inaccessible data are effectively lost until they are made accessible. Moreover, data that are misrepresented or easily misinterpreted can result in conclusions that are even more inaccurate than those that would be drawn if the data were simply lost.

Although it is in the best interest of all who create and use biodiversity data to encourage best practices to protect against data loss, the community still requires additional effective incentives to participate in a shared data environment and to help overcome existing social and cultural barriers to data sharing. Separated silos of data from disparate groups presently dominate the current global infrastructure for biodiversity data. There are some examples of projects working to bring the data out of those silos and to encourage sharing between the various projects. Examples include the Global Biodiversity Information Facility (GBIF) data portal [5] and the Encyclopedia of Life (EOL) [6]. Each of them works to bring together data from their partners [7,8] and makes those data available through their application programming interfaces (APIs). In addition, there are projects, including ScratchPads [9] and LifeDesks [10], that allow taxonomists to focus on their area of expertise while automatically making the data they want to share available to the larger biodiversity community.

Although there is a strong and healthy mix of platforms and technologies, there remains a gap in standards and processes, especially for the 'long tail' of smaller projects [11]. In analyzing existing infrastructure, we see that large and well-funded projects predictably have more substantial investments in infrastructure, making use of not only on-site redundancy, but also remote mirrors. Smaller projects, on the other hand, often rely on manual or semi-automated data backup procedures with little time or resources for comprehensive high availability or disaster recovery considerations.

It is therefore imperative to seek a solution to this data loss and ensure that data are rescued, archived and made available to the biodiversity community. Although there are broad efforts to encourage the use of best practices for data archiving [12-14], citation of data [15,16] and curation [17] as well as large archives that are focused on particular types of biology-related data, including sequences [18-20], ecosystems [21] and observations (GBIF) species descriptions (EOL), none of these efforts are focused on the long-term preservation of biodiversity data. To this end the biodiversity informatics community requires investment in trustworthy processes and infrastructure to mitigate data loss [22] and needs to provide solutions for long-term hosting and storage of biodiversity data. We propose the construction of Biodiversity Data Hosting Centers (BDHCs), which are charged with the task of mitigating the risks presented here by the careful creation and management of the infrastructure necessary to archive and manage biodiversity data. As such, they will provide a future safeguard against loss of biodiversity data.

In laying out our vision of BDHCs, we begin by categorizing the different kinds of biodiversity data that are found in the literature and in various datasets. We then lay out some features and capabilities that BDHC should possess, with a discussion of standards and best practices that are pertinent to an effective BDHC. After a discussion of current tools and approaches for effective data management, we discuss some of the technological and cultural barriers to effective data management and preservation that have hindered the community. We end with a series of recommendations for adopting and implementing data management/preservation changes.

We acknowledge that biodiversity data range widely, both in format and in purpose. Although some authors carefully restrict the use of the term 'data' to verifiable facts or sensory stimuli [23], for the purposes of this article we intend a very broad understanding of the term covering essentially everything that can be represented using digital computers. Although not all biodiversity data are in digital form, we restrict our discussion here to digital data that relate in some way to organisms. Usually the data are created to serve a specific purpose, ranging from ecosystem assessment to species identification to general education. We also acknowledge that published literature is a very important existing repository for biodiversity data, and every category of biodiversity data we discussed may be published in the traditional literature as well as more digitally accessible forms. Consequently, any of the data discussed here may have relevant citations that are important for finding and interpreting the data. However, given that citations have such a rich set of standards and supporting technologies, they will not be addressed here as that would distract from the primary purpose of this paper.

### Categories of data and examples

We start this section with a high-level discussion of the types of data and challenges related specifically to biodiversity data. This is followed by a brief review of various types of existing Internet-accessible biodiversity data sources. Our intent is not to be exhaustive, but rather to demonstrate the wide variety of biodiversity data sources and to point out some of the differences between them. The choice of sites discussed is based on a combination of the authors' familiarity with the particular tools and their widespread use.

Most discussions of the existing data models for managing biodiversity data are either buried in what documentation exists for specific systems, for example, the Architectural View of GBIF's Integrated Publishing Toolkit (IPT) [24] or, alternatively, the data model is discussed in related informally published presentations, for example John Doolan's 'Proposal for a Simplified Structure for EMu' [25]. One example of a data model in the peer-reviewed literature is Rich Pyle's Taxonomer [26] system. However, this is again focused on a particular implementation of a particular data model for a particular system.

Raw observational data are a key type of biodiversity data and include textual or numeric field notes, photographs, video clips, sound files, genetic sequences or any other sort of recorded data file or dataset based on the observation of organisms. Although the processes involved in collecting these data can be complex and may be error prone, these data are generally accepted as the basic factual data from which all other biodiversity data are derived. In terms of sheer volume, raw observational data are clearly the dominant type of data. Another example is nomenclatural data. These data are the relationships between various names and are thus very compact and abstract. Nomenclatural data are generally derived from a set of nomenclatural codes (for example, the International Code of Botanical Nomenclature [27]). Although the correct application of these codes in particular circumstances may be a source of endless debate among taxonomists, the vast majority of these data are not a matter of deep debate. However, descriptions of named taxa, particularly above the species level, are much more subjective, relying on both the historical literature and fundamentally on the knowledge and opinions of their authors. Digital resources often do a poor job of acknowledging, much less representing, this subjectivity.

For example, consider a system that records observations of organisms by recording the name, date, location and observer of the organism. Such a system can conflate objective, raw observational data with a subjective association of that data with a name based on some poorly specified definition of that name. Furthermore, most of the raw observational data are then typically discarded because the observer fails to record the raw data that they used to make the determination or because the system does not provide a convenient way to associate raw data, such as photographs, with a particular determination. Similarly, the person making the identification typically neglects to be specific about what definition of the name they intend. Although this example raises a number of significant questions about the long-term value of such data, the most significant issues for our current purpose relate to combining data from multiple digital resources. The data collected and the way that different digital resources are used are often very different. As a result it is very important to be able to reliably trace the origin of specific pieces of data. It is also important to characterize and document the various data sources in a common framework.

Currently, a wide variety of biodiversity data are available through digital resources accessible through the Internet. These range from sites that focus on photographs of organisms from the general public such as groups within Flickr [28] to large diverse systems intended for a range of audiences such as EOL [6] to very specialized sites focused on the nomenclature of a specific group of organisms, such as Systema Dipterorum [29] or the Index Nominum Algarum [30]. Other sites focused on nomenclature include the International Plant Names Index [31], Index Fungorum [32] and the Catalog of Fishes [33]. Several sites have begun developing biodiversity data for the semantic web [34] including the Hymenoptera Anatomy Ontology [35] and TaxonConcept.org [36]. In addition, many projects include extensive catalogs of names because names are key to bringing together nearly all biodiversity data [37]. Examples include the Catalog of Life [38], WoRMs [39], ITIS [40] and ION [41]. Many sources for names, including those listed above, are indexed through projects such as the Global Names Index [42] and uBio [43].

Some sites focus on curated museum collections (for example, Natural History Museum, London [44], and the Smithsonian Institution [45], among many others). Others focus on descriptions of biological taxa ranging from original species descriptions (the Biodiversity Heritage Library [46] and MycoBank [47]), up-to-date technical descriptions (FishBase [48], the Missouri Botanical Garden [49], and the Atlas of Living Australia [50]), and descriptions intended for the general public (Wikipedia [51] and the BBC Wildlife Finder [52]).

As described above, identifications are often conflated with raw observations or names. However, there are a number of sites set up to interactively manage newly proposed identifications. Examples include iSpot [53], iNaturalist [54], ArtPortalen.se [55] and Nationale Databank Flora en Fauna [56], which accept observations of any organisms, and sites with more restricted domains, such as Mushroom Observer [57], eBird [58] or BugGuide [59]. The GBIF data portal provides access to observational data from a variety of sources, including many government organizations. Many sites organize their data according to a single classification that reflects the current opinions and knowledge of the resource managers. Examples of such sites whose classifications come reasonably close to covering the entire tree of life are the National Center for Biotechnology Information (NCBI) taxonomy [60], the Tree of Life Web [61], the Catalog of Life [38], the World Registry of Marine Species (http://www.marinespecies.org/), and the Interim Register of Marine and Nonmarine Genera [62]. EOL [6] gathers classifications from such sources such and allows the user to choose a preferred classification for accessing its data. Finally, some sites, such as TreeBase [63] and PhylomeDB [64], focus on archiving computer generated phylogenies based on gene sequences.

## Features of biodiversity data hosting centers

Most of the key features of a BDHC are common to the general problem of creating publicly accessible, long-term data archives. Obviously, the data stored in the systems are distinctive to the biodiversity community, such as images of diagnostic features of specific taxa. However, the requirements to store images and the standards used for storing them are widespread. In addition, significant portions of the data models, and thus communication standards and protocols, are distinctive to the biodiversity community. For example, the specific types of relationships between images, observations, taxon descriptions and scientific names.

Government, science, education and cultural heritage communities, among others, are faced with many of the same general challenges that face the biodiversity informatics community when it comes to infrastructure and processes to establish long-term preservation and access of content. The NSF DataNet Program [65] was created with this specific goal in mind. The Data Conservancy [66] and the DataOne Project [67], both funded by DataNet, are seeking to provide long-term preservation, access and reuse for their stakeholder communities. The US National Oceanographic and Atmospheric Administration [68] is actively exploring guidelines for archiving environmental and geospatial data [69]. The wider science and technology community has also investigated the challenges of preservation and access of scientific and technical data [70]. Such investigations and projects are too great to cover in the context of this paper. However, every effort should be made by the biodiversity informatics community to build from the frameworks and lessons learned by those who are tackling these same challenges in areas outside biodiversity.

The primary goal of BDHCs is to substantially reduce the risk of loss of biodiversity data. To achieve this goal they must take long-term data preservation seriously. Fortunately, this need is common to many other areas of data management and many excellent tools exist to meet these needs. In addition to the obvious data storage requirements, data replication and effective metadata management are the primary technologies required to mitigate against the dangers of data loss.

Another key feature of BDHCs is to make the data globally available. Although any website is, in a sense, globally available, the deeper requirements are to make that availability reliable and fast. Data replication across globally distributed data centers is a well-recognized approach to making data consistently available across the world.

Finally, the use and promotion of standards is a key feature for BDHCs. Standards are the key component to enabling effective, scalable data transfer between independently developed systems. The key argument for standards in BDHCs is that they reduce the need for a set of systems that need to communicate with each other to create a custom translator between each pair of systems. Everyone can aim at supporting the standards rather than figuring out how to interoperate with everyone else on a case by case basis. If the number of players is close to the number of standards, then there is no point in standardizing. If each player has its own 'standard', then the total amount of work that has to be done by community goes up as the square of the number of players (roughly speaking, an N^2^ problem, with N being the number of players). If, however, some community standards are used, the amount of work for the community is N×S (with S being the number of standards used in the community). As S approaches N, there is no point in standardizing, but as S becomes much less than N, the total amount of support required by the community to deal with accounting for the various standards decreases. A full account of standards and their importance to robust technical systems is beyond the scope of this article. However, we accept that the use of standards to facilitate data preservation and access is very important for BDHCs. In this context, we present a general overview of common standards used in biodiversity systems in the next section.

## Tools, services and standards

Here we summarize some of the tools, services, and standards that are used in biodiversity informatics projects. Although an exhaustive list is beyond the scope of this article, we attempt to present tools that are widely known and used by most members of the community.

### Tools for replicating data

**TCP/HTTP** protocols allow simple scripting of command-line tools, such as wget or curl, to transfer data. When this is not an option and there is access via a login shell such as OpenSSH, other standard tools can be used to replicate and mirror data. Examples of these are rsync, sftp and scp.

**Peer-to-Peer (P2P)** file-sharing includes technologies such as BitTorrent [71], which is a protocol for distributing large amounts of data. BitTorrent is the framework for the BioTorrents project [72], which allows researchers to rapidly share large datasets via a network of pooled bandwidth systems. This open sharing allows one user to collect pieces of the download from multiple providers, increasing the efficiency of the file transfer while simultaneously providing the downloaded bits to other users. The BioTorrents website [73] acts as a central listing of datasets available to download and the BitTorrent protocol allows data to be located on multiple servers. This decentralizes the data hosting and distribution and provides fault tolerance. All files are then integrity checked via checksums to ensure they are identical on all nodes.

### Tools for querying data providers

**OpenURL **[74] provides a standardized URL format that enables a user to find a resource they are allowed to access via the provider they are querying. Originally used by librarians to help patrons find scholarly articles, it is now used for any kind of resource on the internet. The standard supports linking from indexed databases to other services, such as journals, via full-text search of repositories, online catalogs or other services. OpenURL is an open tool and allows common APIs to access data.

The Biodiversity Heritage Library (BHL)'s OpenURL resolver [75] is an API that was launched in 2009 and continues to offer a way for data providers and aggregators to access BHL material. Any repository containing citations to biodiversity literature can use this API to determine whether a given book, volume, article and/or page is available online through BHL. The service supports both OpenURL 0.1 and OpenURL 1.0 query formats, and can return its response in JSON, XML or HTML formats, providing flexibility for data exchange.

### Tools for metadata and citation exchange

**OAI-PMH** (Open Archives Initiative Protocol for Metadata Harvesting, usually referred to as simply OAI) [76] is an established protocol to provide an application independent framework to harvest metadata. Using XML over HTTP, OAI harvests metadata descriptions of repository records so that servers can be built using metadata from many unrelated archives. The base implementation of OAI must support metadata in the Dublin Core format (a metadata standard for describing a wide range of networked resources), but support for additional representations is available. Once an OAI service is initially harvested, future harvests will check only for new or changed records, making it an efficient protocol and one that is easily set up as a repetitive task that runs automatically at regular intervals.

**CiteBank **[77] is an open access repository for biodiversity publications published by the BHL that allows sharing, categorizing and promoting of citations. Citations are harvested from resources via the OAI-PMH protocol, which seamlessly deals with updates and changes from remote providers. Consequently, all data within CiteBank is also available to anyone via OAI, thus creating a new discovery node. Tools are available that allow users to upload individual citations of whole collections of bibliographies. Open groups may be formed around various categories and can assist in moderating and updating citations.

### Tools for data exchange

**LOCKSS** (Lots of Copies Keep Stuff Safe) [78] is an international community program, based at Stanford University Libraries, that uses open source software and P2P networking technology to map a large, decentralized and replicated digital repository. Using off-the-shelf hardware and requiring very little technical expertise, the system preserves an institution's content while providing preservation for other content - a virtual shared space where all nodes in the network support each other and sustain authoritative copies of the original content. LOCKSS is OAIS (Open Archival Information System) [79] compliant and preserves all genres and formats of web content to preserve both the historical context and the intellectual content. A described 'LOCKSS box' collects content from the target sites using a web crawler similar to the ones that search engines use to discover content. It then watches those sites for changes, allows the content to be cached on the box so as to facilitate a web proxy or cache of the content in case the target system is ever down and has a web-based administration panel to control what is being audited, how often and who has access to the material.

**DiGIR** (Distributed Generic Information Retrieval) [80] is a client/server protocol for retrieving information from distributed resources. Using HTTP to transport Darwin Core XML between the client and server, DiGIR is a set of tools to link independent databases into a single, searchable virtual collection. Although it was initially targeted to deal only with species data, it was later expanded to work with any type of information and was integrated into a number of community collection networks, in particular GBIF. At its core, DiGIR provides a search interface between many dissimilar databases using XML as a translator. When a search query is issued, the DiGIR client application sends the query to each institution's DiGIR provider, which is then translated into an equivalent request that is compatible with the local database. Thus the response can deal with the search even though the details of the underlying database are suppressed, thus allowing a uniform virtual view of the contents on the network. Network speed and availability were major concerns of the DiGIR system; nodes would time out before requests could be processed, resulting in failed queries. The functionality of DiGIR was superceded by the Tapir protocol.

**BioCASE** (The Biological Collection Access Service for Europe) [81] "is a transnational network of biological collections". BioCASE provides access to collection and observational databases by providing an XML abstraction layer in front of a database. Although its search, retrieval and framework is similar to DiGIR, it is nevertheless incompatible with DiGIR. BioCASE uses a schema based on the Access to Biological Collections Data (ABCD) schema [82].

**TAPIR** is a current Taxonomic Database Working Group (TDWG) standard that provides an XML API protocol for accessing structured data. TAPIR extends features of BioCASE and DiGIR by making a more generic method of data interchange. The TAPIR project is run by a task group that oversees the development. The XML request and response for access can be stored in a distributed database. TAPIR combines and extends features of BioCASE and DiGIR to create a more generic means for sharing data. A task group oversees the maintenance of the software as well as the protocol's standard.

Although DiGIR providers are still available, their numbers have diminished since its inception and now number around 260 [83]. BioCASE, meanwhile, has approximately 350 active nodes [84]. TAPIR is being used as the primary collection method by GBIF, who continue to maintain and add functionality to the project. Institutions such as the Missouri Botanical Garden host all three services (DiGIR, BioCASE and TAPIR) to allow ongoing open access to their collections. Many projects will endeavor to maintain legacy tools for as long as there is demand. This support is helpful for systems that have already invested in legacy tools, but it is important to promote new tools and standards to ensure their adoption as improvements in tools and standards are made. In so doing, there needs to be a clear migration path from legacy systems to the new one.

## Distributed computing

### Infrastructure as a service

Cloud infrastructure services provide server virtualization environments as a service. When a user requires a number of servers to store data or run processing tasks, they simply rent computing resources from a provider, who provides the resources from a pool of available equipment. From here, virtual machines can be brought online and offline quickly, with the user paying only for the actual services used or resources consumed. The most well known implementation of this is the Amazon Elastic Compute Cloud (Amazon EC2) [85].

### Distributed processing

Using common frameworks to share processing globally enables researchers to use far more computing power than had been previously possible. By applying this additional computing power, projects can leverage techniques such as data mining to open up new avenues of exploration in biodiversity research. After computational targets are selected, individual jobs are distributed to processing nodes until all work is completed. Connecting disparate system only requires a secure remote connection, such as OpenSSH [86], over the internet.

Apache Hadoop [87] is a popular open source project from Yahoo that provides a Java software framework to support distributed computing. Running on large clusters of commodity hardware, Hadoop uses its own distributed file system (HDFS) to connect various nodes and provides resilience against intermittent failures. Its computational method, map/reduce [88], passes small fragments of work to other nodes in the cluster and directs further jobs while aggregating the results.

Linking multiple clusters of Hadoop servers globally would present biodiversity researchers with a community utility. GBIF developers have already worked extensively with Hadoop in a distributed computing environment, so much of the background research into the platform and its application to biodiversity data has been done. In a post entitled 'Hadoop on Amazon EC2 to generate Species by Cell Index' [89], GBIF generated a 'species per cell index' map of occurrence data across an index of the entire GBIF occurrence record store, where each cell represented an area of one degree latitude by one degree longitude. This map consisted of over 135 million records. This was processed using 20 Amazon EC2 instances running Linux, Hadoop and Java. This map generation was completed in 472 seconds and was used to show that processing tasks could be parsed out over many Hadoop instances to come up with a unified result. Because Hadoop runs in Java it is very simple to provision nodes and link them together to form a private computing network.

### Standards used in the biodiversity community

The biodiversity community uses a large range of data-related standards ranging from explicit data file formats, to extensions to metadata frameworks, to protocols. Larger data objects such as various forms of media, sequence data or other types of raw data are addressed by standardized data file formats, such as JPEG [90], PNG [91], MPEG [92] and OGG [93]. For more specific forms of data, standards are usually decided by the organization governing that data type. The Biodiversity Information Standards [94] efforts are intended to address the core needs for biodiversity data. The metadata standard Darwin Core [95] and the related GBIF-developed Darwin Core Archive [96] file format, in particular, address most of the categories of data discussed above. The current Darwin Core standard is designed to be used in XML documents for exchanging data and is the most common format for sharing zoological data. It is also worth noting that the Darwin Core Archive file format is explicitly designed to address the most common problems that researchers encounter in this space, but not necessarily all of the potential edge cases that could be encountered in representing and exchanging biodiversity data.

The TAPIR [97] standard specifies a protocol for querying biodiversity data stores and, along with DiGIR and BioCASE, provides a generic way to communicate between client applications and data providers. Biodiversity-specific standards for data observations and descriptions are also being developed. For example, the EnvO [98] standard is an ontology similar to Darwin Core that defines a wide variety of environments and some of their relationships.

Community acceptance of standards is an ongoing process and different groups will support various ones at different times. The process for determining a biodiversity specific standard is generally organized by the Biodiversity Information Standards community (organized by TDWG) centered on a working group or mailing list and often has enough exposure to ensure that those with a stake in the standard being discussed will have an opportunity to comment on the proposed standard. This process ensures that standards are tailored to those who eventually use them, which further facilitates community acceptance. Nomenclatural codes in taxonomy, for example, are standardized, and members of this discipline recognize the importance of these standards.

## Barriers to data management and preservation

### Technological barriers

We distinguish between technological and social/cultural barriers to effective data sharing, management and preservation. Technological barriers are those that are primarily due to devices, methods and the processes and workflows that use those devices and methods. Social/cultural barriers are those that arise from both explicit and tacit mores of the larger community as well as procedures, customs and financial considerations of the individuals and institutions that participate in the data management/preservation.

The primary technological cause of loss of biodiversity data is the poor archiving of raw observations. In many cases raw observations are not explicitly made or they are actively deleted after more refined taxon description information has been created from them. The lack of archival storage space for raw observations is a key cause of poor archiving, but simply having a policy of keeping everything is not scalable. At minimum there should be strict controls governing what data are archived. There also must to be a method for identifying data that either are not biodiversity-related or have little value for future biodiversity research. Lack of access to primary biodiversity data can be due to a lack of technical knowledge by the creators of the data regarding how to digitally publish them, and often this is caused by a lack of funding to support publication and long-term preservation. In cases in which data do get published, there remain issues surrounding maintenance of those data sources. Hardware failures and format changes can result in data becoming obsolete and inaccessible if consideration is not given to hardware and file format redundancy.

Another primarily technological barrier to the access and interpretation of biodiversity data is that much of the key literature is not available digitally. Projects such as the BHL are working to address this problem. BHL has already scanned approximately 30 million pages of core biodiversity literature. The project estimates that there are approximately 500 million total pages of core biodiversity literature. Of that, 100 million are out of copyright and are waiting to be scanned by the project.

Interoperability presents a further technological barrier to data access and interpretation. In many cases primary biodiversity data cannot be easily exchanged between a source system and a researcher's system. The standards discussed here help to address this problem, but must be correctly implemented in both the source and destination systems.

Finally, there is no existing general solution for user-driven feedback mechanisms in the biodiversity space. Misinterpretation of biodiversity data is largely the result of incomplete raw observations and barriers to the propagation of data. The process of realizing that two raw observations refer to the same taxon (or have some more complex relationship) takes effort, as does the propagation of that new data. To be successful, such a system would need to be open to user-driven feedback mechanisms that allow local changes for immediate use, which propagate back to the original source of the data. Much of this data propagation could be automated by a wider adoption of standards and better models for the dynamic interchange and caching of data.

### Social and cultural barriers

The social and cultural barriers that cause data loss are well known and not specific to biodiversity data [99]. In addition to outright, unintentional data loss, there are well known social barriers to data sharing. Members of the scientific community can be afraid to share data since this might allow other scientists to publish research results without explicitly collaborating with or crediting the original creator(s) of the data. One approach for addressing this concern is forming data embargoes, in which the original source data are rendered unavailable for a specified period of time relative to its original collection or subsequent publication. In addition, there are no clear standards for placing a value on biodiversity data. This results in radically different beliefs about the value of such data, and in extreme cases can lead scientists to assert rights over data with expensive or severely limited licensing terms.

The financial cost of good archiving and the more general cost of hardware needed, software development and the cost of data creation are all other forms of social barriers to the preservation of biodiversity data [100]. The cost of creating new raw observations continues to drop, but some types of raw observational data are still extremely expensive or otherwise difficult to acquire, especially if significant travel or field work are required to create the data. Similarly, access to museum specimens or living organisms can be crucial to accurately interpreting existing data. There are efficient mechanisms to facilitate such access within the academic sphere, but for scientists outside of the academic world this can be a significant barrier to creating more meaningful data.

Overcoming these barriers will require effective incentives. Ultimately the advantages of having shared, centralized access to the data should serve as its own incentive. This has already happened for genetic sequence data as reflected by the widespread adoption of GenBank [18]. In comparison, biodiversity data have a much greater historical record than sequence data and more importantly a set of legacy conventions that were largely created outside of the context of digital data management. As a result, investment in accurately modeling these conventions and providing easy-to-use interfaces for processing data that conform to these conventions is likely to have greater return in the long run. Providing more direct incentives to the data creators could be valuable as way to get the process started.

### Legal and mandatory solutions

Major US grant funding organizations including the National Institutes of Health [101] and the National Science Foundation [102] are now requiring an up-front plan for data management, including data access and archiving as part of every grant proposal. The National Science Foundation is taking steps to ensure that data from publicly funded research are made public [103]. Such policies, although designed to ensure that data are more accessible, have implications for data archiving. Mandatory data archiving policies are likely to be very effective in raising awareness of issues surrounding data loss and archiving. However, such policies are neither strictly necessary to ensure widespread adoption of data archiving best practices, nor are they a sufficient solution on their own. Adoption of these policies will assist in ensuring that a project's data are available and archivable.

## Recommendations and discussion

Here we recommended five ways in which the community can begin to implement the changes required for sustainable data preservation and access: (1) Encourage the community's use of data standards, (2) promote the public domain licensing of data, (3) establish a community of those involved in data hosting and archival, (4) establish hosting centers for biodiversity data, and (5) develop tools for data discovery.

We prioritize recommendations here from short-term (immediate to 1 year), medium-term (1 to 5 years), to long-term (5 or more years). There is inter-dependency between the recommendations, as having completed one recommendation will make it easier to pursue others.

### 1. Short-term: encourage community use of data standards

The biodiversity community must work in the short term with associations such as the TDWG standards body to strongly promote biodiversity standards in data transfer, sharing and citation. Key players, such as data providers, must ensure that they work to adopt these standards as a short-term priority and encourage other projects to follow suit. The adoption of standards is paramount to the goal of achieving sustainable data preservation and access and paves the way for interoperability between datasets, discovery tools and archiving systems [104]. The processes for standards establishment and community acceptance need to be as flexible and inclusive as possible, to ensure the greatest adoption of standards. Therefore, an open, transparent and easily accessible method for participating in the standards process is important to ensure that standards meet the community's needs. An interesting example is the use of unique identifiers. Popular solutions in the biodiversity community include GUIDs [105], URLs [106] and LSIDs [107]. This debate has not resolved on a single agreed-on standard and it is not clear that it ever will. Therefore, it is important that BDHCs support all such systems, most likely through an internally managed additional layer of indirection and replication of the sources when possible.

### 2. Short-term: promote public domain licensing of content

To minimize the risk of loss, biodiversity data, including the images, videos, classifications, datasets and databases, must all be replicable without asking permission of any rights holders associated with that data. Such data must be placed under as liberal a license as possible or, alternatively, placed into the public domain. This position should be adopted in the short term by existing data providers. To avoid long-term issues, these licenses should allow additional data replication without seeking permission from the original rights holders. A variety of appropriate licenses are available from Creative Commons [108]. 'Non-commercial' licenses should be avoided if possible because this makes it more difficult for the data to be shared and used by sites that may require commercial means to support their projects. In these cases, an open, 'share-alike' license that prevents the end user from encumbering the data with exclusive licenses should be used.

Open source code should be stored in both an online repository, such as github [109] or Google code [110], and mirrored locally at a project's institution for redundancy. Making open source code publicly available is an important and often overlooked step for many projects and must be encouraged by funders, not only for the sake of community involvement in a project, but to allow others to maintain and further develop the software should a project's funding conclude. The use of open source software to support the hosting and development of biodiversity informatics tools and projects should also be encouraged by funders. Using commonplace, open source software lowers the barrier to entry for collaborators, further increasing the chances of a project being maintained after funding has ended.

### 3. Short-term: develop data hosting and archiving community

Encouraging the use of standards and data preservation practices to the wider biodiversity community requires an investment in training resources. In the short term, the community, including projects, institutions and organizations such as GBIF and TDWG, should establish online resources to familiarize researchers with the process of storing, transforming and sharing their data using standards, as well as with the concepts of data preservation and data hosting. Such resources may include screen casts, tutorials and white papers. Even better, intensive, hands-on training programs have proven to be effective in educating domain experts and scientists in technical processes, as can be seen in courses such as the Marine Biology Laboratory's Medical Informatics course, which teaches informatics tools to medical professionals [111]. Funders and organizations such as GBIF should encourage the establishment of such courses for biologists and others working in biodiversity. Partners in existing large-scale informatics projects, such as those of the Encyclopedia of Life or the Biodiversity Heritage Library, as well as institutions such as GBIF regional nodes, would be well placed with the expertise and resources to host such courses. An alternative hands-on method that has been effective is to embed scientists within informatics groups [112].

For hosts of biodiversity data, technical proficiency is important for any information technology project and data archiving and sharing are concepts that most systems engineers are capable of facilitating. Understanding the need for knowledge transfer, a communal group that is comfortable with email and mailing lists is critical for a project's success. Communication between the group can be fostered via the use of open/public wiki, a shared online knowledge base that can be continuously edited as the tools, approaches and best practices change.

**Community input and crowd-sourcing.** Crowd-sourcing [113] is defined as the process of taking tasks that would normally be handled by an individual and opening them up to a larger community. Allowing community input into biodiversity datasets via comments, direct access to the data, adding to the data or deriving new datasets from the original data are all powerful ways of enhancing the original dataset and building a community that cares about and cares for the data. Wikipedia, which allows users to add or edit definitions of any article, is an example of a successful model on a large scale. In the case of biodiversity, there has been success with amateur photographers contributing their images of animals and plants and amateur scientists helping to identify various specimens. Crowd-sourcing is particularly notable as a scalable approach for enhancing data quality. Community input can also work effectively to improve the initial quality of the data and associated metadata [114].

In 2008, the National Library of Australia, as part of the Australian Newspapers Digitization Program (ANDP) and in collaboration with Australian state and territory libraries, enabled public access to selected out-of-copyright Australian newspapers. This service allows users to search and browse over 8.4 million articles from over 830,000 newspapers dating back to 1803. Because the papers were scanned by Optical Character Recognition (OCR) software, they could be searched. Although OCR works well with documents that have consistent typefaces and formats, historical texts and newspapers fall outside of this category, leading to less than perfect OCR. To address this, the National Library opened up the collection of newspapers to crowd-sourcing, allowing the user community the ability to correct and suggest improvements to the OCR text. The volunteers not only enjoyed the interaction, but also felt that they were making a difference by improving the understanding of past events in their country. By March 2010 over 12 million lines of text had been improved by thousands of users. The ongoing results of this work can be seen on the National Library of Australia's Trove site [115]. Biodiversity informatics projects should consider how crowd-sourcing could be used in their projects and see this as a new way of engaging users and the general public.

**Development community to enable biodiversity sharing.** A community of developers that have experience with biodiversity standards should be identified and promoted to assist in enabling biodiversity resources to join the network of BDHC. In general these developers should be available as consultants for funded projects and even included in the original grant applications. This community should also consider a level of overhead funded work to help enable data resources that lack a replication strategy to join the network. A working example of this can be found in tasks groups such as the GBIF/TDWG joint multimedia resources task group, which is working to evaluate and share standards for multimedia resources relevant to biodiversity [116].

**Industry engagement.** Industry has shown, in the case of services such as Amazon's Public Dataset project [117] and Google's Public Data Explorer [118], that there is a great benefit in providing users with access to large public datasets. Even when access to a public dataset is simple, working with that dataset is often not as straightforward. Amazon provides a way for the public to interact with these datasets without the onerous groundwork usually required. Amazon benefits in this relationship by selling its processing services to those doing the research. Engaging industry to host data and provide access methods provides tangible benefits to the wider biodiversity informatics community, in terms of its ability to engage users in developing services on top of the data, with dramatically lower barriers to entry than traditional methods, and from data archival or redundancy perspectives.

### 4. Medium-term: establish hosting centers for biodiversity data

Key players, such as institutions with access to appropriate infrastructure, should act in the medium term to establish biodiversity data hosting centers (BDHCs). BDHCs would mostly probably evolve out of existing projects in the biodiversity data community. These centers will need to focus on collaborative development of software architectures to deal with biodiversity-specific datasets, studying the lifecycle of both present and legacy datasets. Consideration should be made for these data centers to be situated in geographically dispersed locations to provide an avenue for load sharing of data processing and content delivery tasks as well as physical redundancy. By setting up mechanisms for sharing redundant copies of data in hosting centers around the world, projects can take advantage of increased processing power, whereby the project distributes their computing tasks to other participating nodes, allowing informatics teams to more effectively use their infrastructure at times when it would otherwise be underused.

When funding or institutional relationships do not allow mutual hosting and sharing of data, and as a stop gap before specialized data hosting centers become available, third party options should be sought for the backup of core data and related code. By engaging the services of online, offsite backup services, data backup can be automated simply and at a low cost when compared with the capital expense of backup hardware onsite.

When mutual hosting and serving of data is possible, geographically separated projects can take advantage of the low latency and high bandwidth available when data are served from a location local to the user. For example, a project in Europe and a project in Australia who both serve each other's data will serve both projects' data from the closest respective location, reducing bandwidth and latency complications when accessing the data. The creation of specialized data archival and hosting centers, centered on specific data types, would provide for specialized archival of specific formats. For example, with many projects hosting DwC archive files within a central hosting center, the center could provide services to maintain the data and keep it in a current and readable format. By providing this service to a large quantity of data providers, the costs to each provider would be significantly lower than if the data provider were to maintain these formats alone. Such centers would host both standardized, specific formats and provide for a general data store area for raw data that are yet to be transformed into a common format.

In establishing data hosting infrastructure, the community should pay attention to existing efforts in data management standards and hosting infrastructure, such as those being undertaken by the DataONE project, and find common frameworks to build biodiversity data specific solutions.

**Geographically distributed processing.** The sheer size of some datasets often makes it difficult to move data offsite for processing and makes it difficult to conduct research if these datasets are physically distant from the processing infrastructure. By implementing common frameworks to access and process these data, the need to run complex algorithms and tools across slow or unstable internet connections is reduced. It is assumed that more researchers would access data than would have the resources to implement their own storage infrastructure for the data. Therefore it is important for projects to consider the benefits of placing common processing frameworks on top of their data and providing API access to those frameworks in order to provide researchers who are geographically separated from the data access to higher speed processing.

**Data snapshots.** Data hosting centers should track archival data access and updates in order to understand data access patterns. Given the rapid rate of change in datasets, consideration needs to be given to the concept of 'snapshotting' data. In a snapshot, a copy of the state of a dataset is captured at any particular time. The main dataset may then be added to, but the snapshot will always remain static, thereby providing a revision history of the data. An example use case for snapshotting a dataset is an EOL species page. Species pages are dynamically generated datasets comprising user submitted data and aggregated data sources. As EOL data are archived and shared, species pages are updated and modified. Being able to retrieve the latest copy of a dataset may not be as important as retrieving an earlier version of the same dataset, especially if relevant data have been removed or modified. Archival mechanisms need to take snapshotting into consideration to ensure that historical dataset changes can be referenced, located and recovered. This formalized approach to data stewardship makes the data more stable and, in turn, more valuable.

**Data and application archiving.** Although some data exist in standardized formats (for example xls, xml and DwC), others are available only through application-specific code, commonly a website or web service with non-standardized output. Often, these services will store datasets in a way that is optimized for the application code and is unreadable outside the context of this code. Whether data archiving on its own is a sustainable option or whether application archiving is also required needs to be considered by project leaders. Applications that serve data should have a mechanism to export these data into a standard format. Legacy applications that do not have the capability to export data into a standard format need to be archived alongside the data. This can be in a long-term offline archive, where the code is simply stored with the data, or in an online archive where the application remains accessible alongside the data. In both cases, BDHC need to store application metadata, such as software versions and environment variables, both of which are critical to ensuring that the application can be run successfully when required. Re-hosting applications of any complexity can be a difficult task as new software deprecates features in older versions. One solution that BDHC should consider is the use of virtual machines, which can be customized and snapshotted with the exact environment required to serve the application. However, such solutions should be seen as a stop-gap. It should be emphasized that a focus on raw data portability and accessibility is the preferred solution where possible.

**Replicate indexes.** Online data indexes, such as those from GBIF and from the global names index, provide a method for linking online data to objects, in this instance to names. Centrally storing the index enables users to navigate through the indexes to the huge datasets in centralized locations. These data are of particular value to the biodiversity data discovery process as well as being generally valuable for many types of biodiversity research. It is recommended that these index data be replicated widely, and mirroring these indexes should be a high priority when establishing BDHC.

**Tailored backup services.** Given the growing quantity of data and informatics projects, there is scope for industry to provide tailored data management and backup solutions to the biodiversity informatics community. These tools can range from hosting services to online data backup. These services would be designed to deal with biodiversity-related data specifically. By tailoring solutions to the various file formats and standards used in the biodiversity informatics community, issues such as format obsolescence can be better managed. For example, when backing up a mysql file or DarwinCore archive file, metadata associated with that file can be stored in the backup. Such metadata could include the version of DarwinCore or mysql or the date of file creation. These data can be later used when migrating formats in the event of a legacy format becoming obsolete. Such migrations would be difficult for a large number of small projects to consider, so by centralizing this process a greater number of projects will have access to such migrations and therefore the associated data.

### 5. Long-term: develop data discovery tools

The abundance of data made available for public consumption provides a rich source for data-intensive research applications, such as automated markup, text extraction, machine learning, visualizations and digital 'mash-ups' of the data [119]. As a long-term goal for the GBIF secretariat and nodes, as well as biodiversity informatics projects, this process should be encouraged as a natural incentive. Three such uses of these applications are in geo-location, taxon finding and text-mining.

**Services for extracting information.** By extracting place names or geographic coordinates from a dataset, geo-location services can provide maps of datasets and easily combine these with maps of additional datasets, providing mapping outputs such as Google Earth's [120] kmz output. Taxon finding tools can scan large quantities of text for species names, providing the dataset with metadata describing which taxa are present in the data and the location within the data in which each taxon can be found. Using natural language processing, automated markup tools can analyze datasets and return a structured, marked-up version of the data. uBio RSS [121] and RSS novum are two tools that offer automated recognition of species names within scientific and media RSS feeds. By identifying data containing species names, new datasets and sources of data can be discovered. Further development of these tools and integration of this technology into web crawling software will pave the way for automated discovery of online biodiversity data that might benefit from being included in the recommended network of biodiversity data stores. The development of web services and APIs on top of datasets and the investment in informatics projects that harvest and remix data should all be encouraged, both as a means to enable discovery within existing datasets and as an incentive for data owners to publish their data publicly. GBIF, funding agencies and biodiversity informatics projects should extend existing tools while developing and promoting new tools that can be used to enable data discovery. The community should develop a central registry of such tools and, where practical, provide hosting for these tools at data hosting centers.

**Services to enable data push.** As new data are published, there is a lag between the publication time and the harvesting of this data by other services, such as EOL or Google. Furthermore, even after articles are published, data are in most cases available only in HTML format, with the underlying databases rendered inaccessible. Development of services to allow new data to be 'pushed' to online repositories should be investigated, as this would make the new data available for harvesting by data aggregation services and data hosting centers. Once the data are discovered they can then be further distributed via standardized APIs, removing this burden from the original data provider.

Once data aggregators discover new data, they should cache the data and include them in their archival and backup procedures. They should also investigate how best to recover the content provided by a content provider should the need arise. The EOL project follows this recommended practice and includes this caching functionality. This allows EOL to serve aggregated data where the source data are unavailable, while at the same time providing the added benefit of becoming a redundant back-up of many projects' data. This is an invaluable resource should a project suffer catastrophic data loss or go offline.

## Conclusion

As the biodiversity data community grows and standards development and access to affordable infrastructure is made available, some of the above-mentioned challenges will be met in due course. However, the importance of the data and the real risk of data loss suggest that these challenges and recommendations must be faced sooner rather than later by key players in the community. A generic data hosting infrastructure that is designed for a broader scientific scope than biodiversity data may also have a role in access and preservation of biodiversity data. Indeed, the more widespread the data becomes, the safer it will be. However, the community should ensure that the infrastructure and mechanisms it uses are specific enough to biodiversity datasets that they meet both the short-term and long-term needs of the data and community. As funding agencies requiring data management plans becomes more widespread, the biodiversity informatics community will become more attuned to the concepts discussed here.

As the community evolves to take a holistic and collective position on data preservation and access, it will find new avenues for collaboration, data discovery and data re-use, all of which will improve the value of their data. The community needs to recognize and proactively encourage this evolution by developing a shared understanding of what is needed to ensure the preservation of biodiversity data and then acting on the resulting requirements. We see the recommendations laid out in this paper as a step towards that shared understanding.

## Competing interests

The authors declare that they have no competing interests.
